# The Long-Term Cardiovascular Risks of Duloxetine Use in Older Adults: A Retrospective Medical Record-Based Adverse Drug Reaction Assessment

**DOI:** 10.3390/jcm13247595

**Published:** 2024-12-13

**Authors:** Yuqi Cui, Sayed Aliul Hasan Abdi, Jeanne Wei, Gohar Azhar

**Affiliations:** 1 Department of Geriatrics, Donald W. Reynolds Institute on Aging, University of Arkansas for Medical Sciences, Little Rock, AR 72205, USA; ycui@sbrmc.org (Y.C.); hasansayed@uams.edu (S.A.H.A.); weijeanne@uams.edu (J.W.); 2 Internal Medicine Residency Program, St. Bernards Medical Center, Jonesboro, AR 72401, USA

**Keywords:** duloxetine, cardiovascular risk, adverse drug reactions, older individuals

## Abstract

**Background:** Duloxetine, a Serotonin–Norepinephrine Reuptake Inhibitor (SNRI), is frequently used to treat diabetic peripheral neuropathy, depression, and fibromyalgia. However, its long-term cardiovascular implications in older individuals remain underexplored, particularly in those with pre-existing cardiovascular diseases. This medical record assessment aimed to evaluate the potential cardiovascular risks of duloxetine use in older persons after prolonged use. **Methods:** We evaluated adverse drug reactions (ADRs) using six medical records from elderly individuals (aged 70–79) with cardiovascular comorbidities who received duloxetine (≥60 mg daily) for anxiety, depression, and chronic pain. ADRs were assessed using the Naranjo ADR Probability Scale, the Modified Hartwig and Siegel Severity Scale, and the Karch and Lasagna Algorithm. Clinical outcomes were assessed before and after duloxetine dose reduction or withdrawal. **Results:** All the patients had cardiovascular-related ADRs, such as peripheral cyanosis, vasoconstriction, atrial fibrillation, and hypertensive episodes. Five of the six patients experienced mild cognitive impairment [Montreal Cognitive Assessment (MoCA) scores of 11–24/30]. A positive dechallenge (symptom resolution) was observed in all medical records after decreasing or discontinuing duloxetine. It is interesting to note that four medical records demonstrated significant improvement in cyanosis, blood pressure, and anxiety after decreasing or discontinuing duloxetine use. There was no rechallenge in this study. The causality was considered probable (Naranjo Scale), and ADRs were categorized as moderately severe (Hartwig and Siegel Scale) in all the medical records. However, with adequate monitoring, the ADRs were considered preventable (Schumock and Thornton Scale). **Conclusions:** Long-term duloxetine use could cause significant cardiovascular problems in older individuals, particularly those who already have cardiovascular difficulties. Regular monitoring of cardiovascular function and early steps such as dose adjustment or drug withdrawal of duloxetine may reduce the prognosis of ADRs. More studies are required to create safer treatment strategies for managing depression and anxiety in older people with cardiovascular issues.

## 1. Introduction

Duloxetine is a versatile Serotonin–Norepinephrine Reuptake Inhibitor (SNRI) that is approved for depression, diabetic peripheral neuropathy, and fibromyalgia [1]. With its balanced serotonin and norepinephrine affinity, duloxetine offers pain relief and mood enhancement [1]. Despite in vitro studies suggesting a potential inhibitory effect on cardiac sodium channels, clinical trials in healthy subjects and geriatric populations with depression or generalized anxiety disorder (GAD) with short-term duloxetine treatment demonstrated either minimal cardiac effects or a tendency towards orthostatic hypotension in high-risk patients [1]. However, the cardiovascular safety profile of duloxetine remains a topic of therapeutic and clinical interest, particularly in older individuals, where comorbidities such as hypertension and cardiac disease are more common. Although in vitro studies suggest that the blockage of cardiac sodium channels may predispose patients to arrhythmias, real-world data and short-term clinical trials have revealed a minimal prevalence of serious cardiac side-effects. The most often documented cardiovascular side-effects include temporary elevations in blood pressure and orthostatic hypotension, particularly at higher doses or in patients with previous cardiovascular risk factors [2,3,4,5]. This emphasizes the significance of constant monitoring, especially during the initiation and titration periods of treatment, to reduce possible hazards in vulnerable populations.

Therefore, our study aimed to identify the risk of ADRs from duloxetine in older individuals.

## 2. Methods

This study was conducted as a retrospective review of medical records.

Since only medical records were reviewed and all data were de-identified, informed consent was not required. In this retrospective medical record assessment, we describe patients in the geriatric age group who had been seen in the clinic for memory problems and had been on duloxetine for an extended period of time. The study was approved by the Institutional Review Board of the University of Arkansas for Medical Sciences (IRB # 133328).

The ADR assessment was conducted using the Naranjo ADR Probability Scale, the Modified Hartwig and Siegel Severity Scale, and the Karch and Lasagna Algorithm [6,7,8,9]. The arbitrators Wei J and Azhar G were contacted for final ADR scale score confirmation, and the ADR scores were blinded to each reviewer. The descriptions of the utilized scales are provided in Supplementary File S1.

### 2.1. Medical Record I

A 73-year-old female with a 20-year history of fibromyalgia, anxiety, depression, hypertension, paroxysmal supraventricular tachycardia (PSVT) and status post-cardiac pacemaker, presented for memory assessment. The patient’s anxiety and depression had been managed with escalating doses of duloxetine (60 mg daily) for a decade. In spite of frequent emergency room visits for anxiety, palpitations, and PSVT, a definitive diagnosis of atrial fibrillation had been overlooked, leading to a lack of anticoagulation. In fact, during the past decade, her complaints of palpitations and tachycardia had often been attributed to anxiety and depression, and the dose of duloxetine had been increased.

A recent consultation with a cardiologist had revealed atrial fibrillation, and the patient was started on apixaban. Her new medication regimen included sotalol, diltiazem, gabapentin, ranitidine, and hydrochlorothiazide. The dose of 60 mg of duloxetine was continued. The patient sought consultation for memory problems in a geriatric memory clinic. The neurological examination was largely unremarkable, but there were signs of vasoconstriction with mild peripheral cyanosis of her fingers, prompting further investigations. Her Montreal Cognitive Assessment (MoCA) score was 24/30. A diagnosis of mild cognitive impairment (MCI) with anxiety and mild depression was made. The etiology of the MCI was primarily vascular due to atrial fibrillation.

#### 2.1.1. Investigations

An FDG18 PET CT indicated mild hypometabolism in the left inferior–parietal lobe, suggesting vascular damage. The patient’s medication regimen, notably, with duloxetine acting as a sympathomimetic, was implicated in contributing to vasoconstriction, anxiety episodes, PSVT, and cognitive issues.

#### 2.1.2. Management and Outcome

The management in the geriatric clinic focused on the gradual discontinuation of duloxetine, with a transition to sertraline. Vasoconstriction and cyanosis were resolved within 3–4 weeks. Subsequent follow-ups demonstrated marked improvement in anxiety, ability to focus, and energy levels.

### 2.2. Medical Record II

A 75-year-old male with a history of hypertension, Parkinson’s disease, essential tremor, anxiety, and depression had been on duloxetine 60 mg/day for an extended period. He initially presented with cyanosis and cold sensations in both feet, with a blood pressure of 140–150/70–80 mmHg. His MoCA score was 20/30, and a Dopamine Transporter (DAT) scan showed dopamine deficiency that was consistent with atypical Parkinson’s disease.

His medication regimen included propranolol for hypertension and duloxetine for depression, which he had been taking for about six years. Further evaluation led to a diagnosis of MCI due to vascular cognitive decline and dopamine deficiency.

It appeared probable that duloxetine might be contributing to the peripheral vasoconstriction and cold sensation in the peripheries, and hence, the patient’s duloxetine was gradually tapered off, and he was transitioned to sertraline. Following the discontinuation of duloxetine, the symptoms of cyanosis and cold sensations in the feet were resolved. Additionally, his blood pressure improved to 110–130/70–80 mmHg.

### 2.3. Medical Record III

A 79-year-old male with aortic valve stenosis, hypertension, and coronary artery disease (CAD) presented with predominant vascular cognitive decline. His MoCA score was 11/30. In addition, he suffered from anxiety and fatigue. He also felt cold and sometimes had a bluish tinge to his fingers and toes.

For anxiety and fatigue, he had been taking duloxetine for about a year, and during the past six months, his dose had been 120 mg daily. He had also been on losartan 100 mg daily and amlodipine 5 mg daily for the past 6 years. His anxiety and symptoms of cold and discolored peripheries were all consistent with vasoconstriction. Therefore, duloxetine was tapered and reduced to 20 mg over five months. Just by reducing the duloxetine dose, the patient’s blood pressure decreased from 170–160/80–90 mmHg to 110–120/70–80 mmHg. In addition, his anxiety episodes abated, as did the cold and cyanotic fingers and toes.

### 2.4. Medical Record IV

A 70-year-old female with a PMH of HTN, perforated gastric ulcer, and transient ischemic attacks developed atrial fibrillation post-COVID-19 and was on sotalol 40 mg twice daily. She was not anticoagulated because of a history of gastric ulcer. In addition, she suffered from anxiety and dizziness and experienced frequent falls. Her memory testing was compatible with mild cognitive impairment.

She had been prescribed duloxetine 60 mg daily for anxiety and depression and had been taking it for about 7 months. However, due to concerns about the long-term use of duloxetine and its potential effects on atrial fibrillation and transient ischemic attacks, the decision was made to taper off duloxetine. The reduction in and discontinuation of duloxetine over nine months resulted in a significant reduction in anxiety, although her blood pressure and atrial fibrillation were not impacted.

### 2.5. Medical Record V

A 75-year-old female with a PMH of HTN, rheumatic aortic valve insufficiency, PAT, major depressive disorder (MDD), and fibromyalgia had been on duloxetine 60 mg daily for MDD, fibromyalgia, and severe arthritis pain for nine years. She had also been taking lisinopril 5 mg daily and metoprolol tartrate 25 mg twice a day for HTN. She had visited the ER multiple times due to various cardiovascular symptoms, including some chest pain with accelerated hypertension. Her troponin levels were normal. Cardiovascular magnetic resonance and single-photon emission computed tomography tests showed no reversible perfusion defects. A coronary angiography showed a normal coronary angiogram, and an echocardiogram revealed no significant abnormalities.

Duloxetine was reduced to 30 mg daily, and she has had no more ER visits due to cardiovascular symptoms. However, she still experiences some PSVT and has continued taking apixaban.

### 2.6. Medical Record VI

A 70-year-old female with a PMH of HTN, atherosclerosis, and fibromuscular dysplasia had experienced back pain for a long time. She was placed on duloxetine 30 mg daily for 6 years for her back pain, but this caused an increase in her blood pressure. Her baseline blood pressure was 140–150/70–80 mmHg, which accelerated and went over 180–200/80–90 mmHg, leading her to visiting the ER several times due to hypertensive urgency. She was also on propranolol 10 mg three times a day for HTN.

Duloxetine was gradually discontinued, and her blood pressure gradually decreased to 120–130/70–80 mmHg without any further ER visits for hypertensive urgency.

#### 2.6.1. Adverse Reaction Assessment

All relevant data, including each patient’s age, gender, medications received prior to the onset of reactions, route of administration, and frequency of administration, were collected. Patients were closely monitored and assessed daily during their hospital stay for possible reactions [10,11].

#### 2.6.2. Naranjo Adverse Drug Reaction Probability Scale

Medical records: Each medical record in the series reported improvement after reducing or discontinuing duloxetine, suggesting a strong temporal relationship and positive dechallenge.

#### 2.6.3. Application

Temporal relationship: Present in all medical records.

Dechallenge: The symptoms improved once the duloxetine dose was reduced (positive dechallenge).

Rechallenge: Not tested.

Possible alternative causes: Present (underlying cardiovascular concerns), although ADRs are more likely due to the persistent improvement upon duloxetine withdrawal.

Naranjo Score: Likely to be in the “probable” category (score 5–8), as dechallenge occurred, but no rechallenge was performed.

Modified Hartwig and Siegel Severity Assessment Scale

Medical record I: The patient’s atrial fibrillation and vasoconstriction were resolved with discontinuation and were likely moderate (Level 3–4), because they required an intervention, but there was no report of permanent damage.

Medical record II: Cold extremities, cyanosis, and cognitive decline were resolved—moderate (Level 3–4).

Medical record III: Vasoconstriction and anxiety were improved—moderate (Level 3–4).

Medical record IV: No cardiovascular impact was recorded, but the patient’s anxiety was decreased—mild (Level 1–2).

Medical record V: Recurrent cardiovascular symptoms requiring emergency visits were moderate (Level 4–5), as they required frequent hospital visits and interventions.

Medical record VI: Hypertensive urgency requiring emergency visits—moderate (Level 4–5).

Overall Severity: Most of the ADR assessments based on the included medical record were classified as moderately severe, because they required hospitalization or a modification in pharmacological regimen.

#### 2.6.4. Modified Schumock and Thornton Scale

##### Criteria for Preventability

Medical records I–VI: Given duloxetine’s known cardiovascular risks and the possibility of vasoconstriction in older individuals with comorbidities, these ADRs may have been “possibly preventable”. Appropriate ADR monitoring may have led to a reduction in long-term effects.

Outcome: Possibly preventable, in most medical records, due to the known risks of duloxetine in elderly individuals with prior cardiovascular problems. The ADRs may have been preventable in the majority of occurrences.

##### Karch and Lasagna’s Algorithm

Temporal Relationship: In all medical records, all ADRs developed with long-term duloxetine use and were resolved after cessation.

Dechallenge: A positive dechallenge was noted in all patients, and symptoms improved after lowering or discontinuing duloxetine use.

Possible explanations: While cardiovascular comorbidities persisted, the consistent resolution of symptoms post-duloxetine reduction supports a causal relationship with duloxetine.

Karch and Lasagna’s Conclusion: It is likely that “definite” or “probable” causality existed for duloxetine-induced ADRs due to the evident timeline and improvement upon discontinuation.

## 3. Summary

1. Naranjo Scale: Probable ADRs are due to a clear temporal link and positive dechallenge.
2. Hartwig and Siegel: Most medical records indicate a moderate degree of severity (moderately severe), with some requiring an emergency visit.
3. Schumock and Thornton: Possibly preventable, due to known cardiovascular risks in older adults.
4. Karch and Lasagna: Definite or probable ADRs, with symptom relief following drug cessation.

This assessment demonstrates that duloxetine played a substantial role in the observed ADRs and indicates that duloxetine usage in older people requires careful monitoring. A comparative assessment is presented in Table 1.

## 4. Discussion

This study emphasizes the need for a comprehensive evaluation of older adults receiving duloxetine. While numerous clinical trials present a favorable cardiovascular profile for duloxetine, with slight increases in diastolic blood pressure and heart rate and minimal orthostatic hypotension risk, these trials typically range from 9 to 52 weeks (see Table 2).

**Figure 1:**
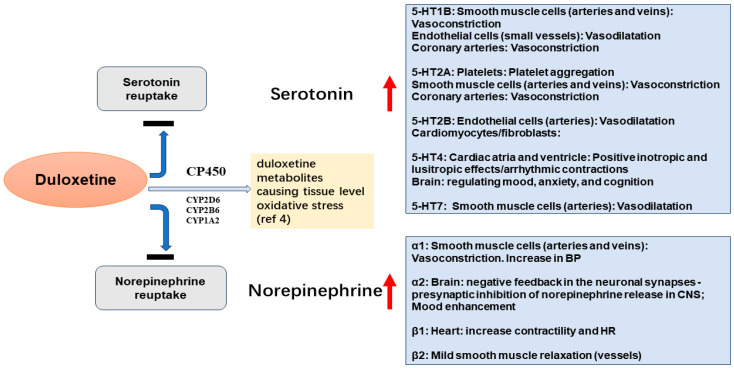
Physiological impacts and potential toxic effects of duloxetine on the brain and cardiovascular system. Red arrows denote increase in Serotonin and Norepinephrine enhancing the cellular and physiological responses outlined in the adjacent boxes, respectively.

The cases in our study report histories of duloxetine usage ranging from 7 months to approximately 10 years, during which patients developed various cardiovascular diseases, including AF, vascular dementia, and peripheral vascular diseases (PVDs). Some symptoms, such as HTN and PVD, significantly improved after the discontinuation of duloxetine. However, non-reversible symptoms such as vascular dementia and AF remained despite the tapering and/or discontinuation of duloxetine. Early discontinuation of duloxetine could have prevented these severe consequences. Cardiomyocytes and neurons are both terminally end-differentiated, highly specialized cells, and norepinephrine-mediated vasoconstriction can damage both cardiac and cerebral tissues (Figure 1).

Thus, careful consideration of the cardiovascular side-effects and their potential impact on cognitive function is crucial when using duloxetine in older adults. Timely diagnosis and management, including medication adjustments, can significantly improve outcomes in this vulnerable population.

Duloxetine is a versatile Serotonin–Norepinephrine Reuptake Inhibitor (SNRI) that is approved for depression, diabetic peripheral neuropathy, and fibromyalgia [1]. The combination of serotonin and norepinephrine upregulates the mood, and the increase in norepinephrine improves focus and energy. However, norepinephrine could act as a double-edged sword in older adults and might have a negative clinical impact with long-term use in those with a high cardiovascular risk score or vascular cognitive impairment (Figure 1).

Duloxetine inhibits CYP2D6, 2B6, and 1A2 enzymes, potentially affecting drug metabolism, which is a concern in older adults because of the reduced and altered drug metabolism and high prevalence of polypharmacy in the geriatric population [1]. Interestingly, more recent in vivo studies using duloxetine in rodents for a duration equivalent to two years in humans showed significant oxidative damage to the cardiac muscles, with vacuolation of cardiomyocytes, intracellular edema, and contraction band necrosis of sarcomeres [4]. These oxidative injury changes were observed more in female rats, which could potentially be explained by the higher levels of protective CYP1A2 enzyme in the hearts of females vs. males [4].

Despite in vitro studies suggesting a negative impact of duloxetine on cardiac sodium channels and in vivo rodent data demonstrating cardiac oxidative stress, clinical trials in healthy older adults have reported minimal cardiovascular effects [1]. In older adults, duloxetine has been described to have a relatively low-risk side-effect profile, with slight increases in diastolic blood pressure and heart rate and minimal orthostatic hypotension risk [1]. Clinical trials in geriatric populations with depression or generalized anxiety disorder (GAD) also reveal negligible cardiovascular concerns [1]. In diabetic patients with peripheral neuropathy, duloxetine has demonstrated cardiovascular safety, with only slight increases in heart rate and no QTc abnormalities [1].

A systematic review and meta-analysis found that while duloxetine minimally increased the heart rate (HR) and diastolic blood pressure (DBP), these changes were considered clinically insignificant. Despite this, the study noted that several cardiovascular-related adverse events were identified, suggesting the need for further investigation into the cardiovascular risks associated with duloxetine [2]. One case highlighted a 45-year-old female who, three days after initiating duloxetine treatment for depressive disorder without a history of hypertension, experienced severe headaches and a blood pressure of 170/110 mmHg. Discontinuation of duloxetine and initiation of escitalopram resolved her hypertension [3]. Another case involved an 82-year-old woman with a history of hypertension and bipolar disorder who developed acute left upper limb weakness and facial paralysis after starting duloxetine. She also experienced severe headaches, transient loss of consciousness, and a hypertensive crisis. Imaging revealed brain abnormalities that were consistent with posterior reversible encephalopathy syndrome (PRES). Treatment with anticonvulsants and blood pressure management led to a full recovery, suggesting a diagnosis of PRES induced by duloxetine [5].

Our study highlights the potential long-term cardiovascular, as well as cognitive, risks associated with duloxetine use in older adults, which is in contrast with the findings of current clinical trials that focus on short-term usage. This emphasizes the critical need to recognize and address these long-term effects in older populations, who are already prone to oxidative stress with aging. It is possible that duloxetine might not be the best drug for the treatment of depression and chronic pain in older adults, especially if they suffer from tachyarrhythmias or have angina or peripheral vascular disease [18]. However, a report from Wernicke et al. [19] highlighted that duloxetine did not significantly affect cardiovascular parameters when compared with placebo, indicating its relative safety. In addition, a study by Zhang et al. [12] found no significant alterations in ventricular repolarization among patients taking duloxetine, further supporting its cardiovascular safety. Switching from duloxetine to sertraline, an SSRI, has been documented in numerous cases, especially when patients experience adverse side-effects or have particular cardiovascular concerns. Sertraline is noted for its favorable cardiovascular safety profile, making it a potentially suitable alternative for specific patient groups [20,21,22]. Further research is imperative to ensure the safety of duloxetine in managing depression or chronic pain and its impact on cardiovascular and cognitive outcomes, especially in the geriatric population.

In addition, all the detected ADRs were evaluated using ADR scales. The key observations based on the ADR scales used in this study provide insights into the relationship between duloxetine and its adverse effects in older individuals.

## 5. Recommendations for Improvement

Improved Monitoring: it is recommended that older patients on duloxetine have their cardiovascular functions thoroughly evaluated.

Plan for Personalized Treatment: these findings imply that the physician should carry out a more thorough assessment of the patients’ cardiovascular risks before administering duloxetine, particularly in those with comorbidities.

Methods for Prevention: Given the Schumock and Thornton Scale’s finding that these ADRs are potentially preventable, methods such as regular blood pressure monitoring, dosage modifications, and ECGs may help reduce risks [22,23].

## 6. Limitations

With only six medical records, our observations are limited in scope and cannot show causation or generalizability. However, our intent was to provide clinically relevant insights that highlight potential risks in real-world geriatric populations, particularly those with complicated comorbidities. As for the temporal relationship between duloxetine initiation and adverse drug reactions (ADRs), we aimed to document this link to the best possible extent based on patient records and clinical observations. However, given the retrospective nature of this series and some patients’ multifactorial risk factors, the precise timings may not always be clear, which we acknowledge as a limitation. The study could not assess the entirety of the problem, as the incidence rate of adverse drug reactions cannot be determined due to the study design.

In our retrospective analysis, a rechallenge test was not feasible. However, we understand that a rechallenge test may provide stronger evidence of causality, although it would need to be performed in a prospective clinical trial with due consideration for potential risks, particularly in older individuals with multiple comorbidities.

## 7. Conclusions

Limiting long-term duloxetine use and utilizing lower doses in older people, especially patients with cardiovascular problems, may have a favorable impact in mitigating both cardiovascular and cognitive complications. The ADR analysis carried out in this study emphasizes the importance of comprehensive drug monitoring of duloxetine. Clinicians must exercise caution in recognizing and managing the potential ADRs of SNRI drugs, particularly duloxetine, while tailoring the duloxetine dose to improve the overall health of older persons. In our study, patients presented with a variety of signs and symptoms that could be attributed to adrenergic side-effects of duloxetine on the cardiovascular system. Additionally, these symptoms were ameliorated following the lowering of the dose of duloxetine or discontinuing its use. Hence, our study highlights the potential for long-term exposure to increased cardiovascular risks for older adults receiving duloxetine, which is contrary to the relative safety observed in clinical trials of shorter durations. This underscores the importance of recognizing and addressing the adverse effects of duloxetine in older populations. Further research is warranted to ensure duloxetine’s appropriate use and safety, especially for older adults and in the context of cardiovascular comorbidities.

## Figures and Tables

**Table 1:** Comparison of the adverse drug reactions (ADRs) of duloxetine in the medical records.

Scale	Description	Medical Record I (73 F)	Medical Record II (75 M)	Medical Record III (79 M)	Medical Record IV (70 F)	Medical Record V (75 F)	Medical Record VI (70 F)
Naranjo Adverse Drug Reaction Probability Scale	Assesses the probability of a drug causing an ADR based on criteria such as temporal relationship, dechallenge, and alternative causes.	Probable (score: 6–7)	Probable (score: 6–7)	Probable (score: 6–7)	Probable (score: 5–6)	Probable (score: 6–7)	Probable (score: 6–7)
Modified Hartwig and Siegel Severity Assessment Scale	Assesses the severity of an ADR based on the level of intervention required and outcomes.	Moderate (Level 3–4)	Moderate (Level 3–4)	Moderate (Level 3–4)	Mild (Level 1–2)	Moderate (Level 4–5)	Moderate (Level 4–5)
Modified Schumock and Thornton Scale	Assesses whether an ADR was preventable based on the appropriateness of drug use and monitoring.	Possibly preventable	Possibly preventable	Possibly preventable	Possibly preventable	Possibly preventable	Possibly preventable
The Karch and Lasagna’s Algorithm	Evaluates ADR causality by examining temporal relationships, dechallenge, and alternative explanations.	Definite or probable causality	Definite or probable causality	Definite or probable causality	Definite or probable causality	Definite or probable causality	Definite or probable causality

M: Male; F: female.

**Table 2:** Clinical trials with duloxetine examining cardiovascular side-effects and safety.

Subject Information	Intervention	Outcome	Ref.
Age range, 19–47 yrs; *n* = 117; females; healthy.	Duloxetine dose was increased over 20 days from 60 mg bid to 200 mg bid. A single dose of moxifloxacin 400 mg was used as a control.	Duloxetine did not affect ventricular repolarization.	Zhang et al., 2007 [12]
Age range, 18–65 yrs; *n* = 378 males; *n* = 761 females; depression.	Duloxetine dose range 40–120 mg/d for 8–9 weeks.	Duloxetine had modest effects on heart rate and blood pressure and no meaningful effects on EKGs in the relatively healthy group of clinical trial subjects.	Thase et al., 2005 [13]
Age range, 65–87 yrs; *n* = 29 males; *n* = 72 females; MDD.	Duloxetine 40–60 mg bid for up to 52 weeks.	The average change in blood pressure was less than 2.0 mmHg.	Wohlreich et al., 2004 [14]
Age range, 65–90 yrs; *n* = 82 males; *n* = 125 females; MDD.	Duloxetine 60 mg daily for 8 weeks.	The study reported that duloxetine is safe and well tolerated. No major concerns about cardiovascular safety or falls in older patients were reported.	Raskin et al., 2008 [15]
Age range, 65–89.5 yrs; *n* = 39 males; *n* = 56 females; MDD.	Duloxetine 60 or 120 mg daily for 24 weeks.	Orthostatic BP changes were marginally significant at 24 weeks. Resting HR increased significantly at 24 weeks. One serious adverse event involved a hip fracture due to hypotension.	Robinson et al., 2013 [16]
Mean age, 71.4 yrs; *n* = 37 males; *n* = 114 females; GAD.	Duloxetine 30 mg daily for the first 2 weeks, then increased up to 90–120 mg daily for another 8 weeks.	Clinically non-significant changes in systolic blood pressure and ECG markers. Slight increase in diastolic blood pressure and HR.	Huang et al., 2011 [17]

MDD: major depressive disorder; GAD: generalized anxiety disorder.

## Data Availability

The data presented in this article are not readily available because this was a retrospective case series study of medical records only. Requests or questions regarding it should be directed to the corresponding author.

## Supplementary Materials

The following supporting information can be downloaded at: https://www.mdpi.com/article/10.3390/jcm13247595/s1.
