# Supplementary material for: The Long-Term Cardiovascular Risks of Duloxetine Use in Older Adults: A Retrospective Medical Record-Based Adverse Drug Reaction Assessment
Source: J Clin Med. 2024 Dec 13;13(24):7595. doi: 10.3390/jcm13247595 (PMC11676122; doi:10.3390/jcm13247595)
Supplement: Supplementary file 1 [file jcm-13-07595-s001.zip › Supplementary File S1.pdf]

Naranjo Adverse Drug Reaction Probability Scale

Question	Yes	No	Don't know
1. Are there previous *conclusive* reports on this reaction?	+1	0	0
2. Did the adverse event appear after the suspected drug was administered?	+2	-1	0
3. Did the adverse reaction improve when the drug was discontinued or a *specific* antagonist was administered?	+1	0	0
4. Did the adverse event reappear when the drug was re-administered?	+2	-1	0
5. Are there alternative causes (other than the drug) that could on their own have caused the reaction?	-1	+2	0
6. Did the reaction reappear when a placebo was given?	-1	+1	0
7. Was the drug detected in blood (or other fluids) in concentrations known to be toxic?	+1	0	0
8. Was the reaction more severe when the dose was increased or less severe when the dose was decreased?	+1	0	0
9. Did the patient have a similar reaction to the same or similar drugs in *any* previous exposure?	+1	0	0
10. Was the adverse event confirmed by any objective evidence?	+1	0	0
Total score			

Adapted from - Naranjo CA, Busto U, Sellers EM, Sandor P, Ruiz I, Roberts EA, et al. A method for estimating the probability of adverse drug reactions. Clinical Pharmacology & Therapeutics. 1981;30(2):239–45.

Modified Hartwig and Siegel Severity Assessment Scale

Level	Description
1	The ADR requires no change in treatment with the suspected drug
2	The ADR requires the suspected drug to be withheld, discontinued or otherwise changed. No antidote or other treatment is required. There is no increase in length of hospital stay
3	The ADR requires that the suspected drug be withheld, discontinued or otherwise changed, and/or an antidote or other treatment is required. There is no increase in length of hospital stay
4	Level 4a - Any level 3 ADR that increases the length of hospital stay by at least one day<br><br>Level 4b – The ADR is the reason for admission
5	Any level 4 ADR that requires intensive medical care
6	The ADR causes permanent harm to the patient
7	The ADR either directly or indirectly leads to the death of the patient

Original version of the scale - Hartwig SC, Siegel J, Schneider PJ. Preventability and severity assessment in reporting adverse drug reactions. American journal of hospital pharmacy. 1992;49(9):2229–32.

Modified Schumock and Thornton scale

Questions for assessment of preventability

Definitely preventable

1. Was there a history of allergy or previous reactions to the drug?
2. Was the drug involved inappropriate for the patient's clinical condition?
3. Was the dose, route or frequency of administration inappropriate for the patient's age, weight or disease state?
4. Was a toxic serum drug concentration (or laboratory monitoring test) documented?
5. Was there a known treatment for the Adverse Drug Reaction?

Probably preventable

6. Was required Therapeutic drug monitoring or other necessary laboratory tests not performed?
7. Was a drug interaction involved in the ADR?
8. Was poor compliance involved in the ADR?
9. Were preventative measures not prescribed or administered to the patient?

Not preventable

If all above criteria not fulfilled

Original version of the scale - Schumock GT, Thornton JP. Focusing on the preventability of adverse drug reactions. Hospital pharmacy. 1992;27(6):538.

The Karch and Lasagna's Algorithm

**Question**	**Yes**	**No**	**Do not know**	**Score**
1.Are there previous conclusive reports on this reaction?	+1	0	0	-
2. Did the adverse event appear after the suspected drug was administered?	+2	-1	0	-
3. Did the adverse event improve when the drug was discontinued or a specific antagonist was administered?	+1	0	0	-
4. Did the adverse event reappear when the drug was readministered?	+2	-1	0	-
5.Are there alternative causes that could on their own have caused the reaction?	-1	+2	0	-
6. Did the reaction reappear when a placebo was given?	-1	+1	0	-
7. Was the drug detected in blood or other fluids in concentrations known to be toxic?	+1	0	0	-
8. Was the reaction more severe when the dose was increased or less severe when the dose was decreased?	+1	0	0	-
9. Did the patient have a similar reaction to the same or similar drugs in any previous exposure?	+1	0	0	-
10. Was the adverse event confirmed by any objective evidence?	+1	0	0	-
			Total Score: define>8;<br>probable 5-8;<br>possible 1-4;<br>doubtful 0	
